# *Acacia catechu* (L.f.) Willd.: A Review on Bioactive Compounds and Their Health Promoting Functionalities

**DOI:** 10.3390/plants11223091

**Published:** 2022-11-14

**Authors:** Monika Kumari, Radha, Manoj Kumar, Baohong Zhang, Ryszard Amarowicz, Sunil Puri, Ashok Pundir, Sonia Rathour, Neeraj Kumari, Deepak Chandran, Abhijit Dey, Niharika Sharma, Sureshkumar Rajalingam, Pran Mohankumar, Surinder Sandhu, Nutan Pant, Raja Priya Ravichandran, Marimuthu Subramani, Kunjammal Pandi, Muthamilselvan Muthukumar, Gokhan Zengin, Mohamed Mekhemar, Jose M. Lorenzo

**Affiliations:** 1 School of Biological and Environmental Sciences, Shoolini University of Biotechnology and Management Sciences, Solan 173229, India; 2 Chemical and Biochemical Processing Division, ICAR—Central Institute for Research on Cotton Technology, Mumbai 400019, India; 3 Department of Biology, East Carolina University, Greenville, NC 27858, USA; 4 Institute of Animal Reproduction and Food Research, Polish Academy of Sciences, 10-748 Olsztyn, Poland; 5 School of Mechanical and Civil Engineering, Shoolini University of Biotechnology and Management Sciences, Solan 173229, India; 6 Department of Veterinary Sciences and Animal Husbandry, Amrita School of Agricultural Sciences, Amrita Vishwa Vidyapeetham University, Coimbatore 642109, India; 7 Department of Life Sciences, Presidency University, 86/1 College Street, Kolkata 700073, India; 8 Department of Agronomy, Amrita School of Agricultural Sciences, Amrita Vishwa Vidyapeetham University, Coimbatore 642109, India; 9 School of Agricultural Sciences, Karunya Institute of Technology and Sciences, Coimbatore 641114, India; 10 Department of Plant Breeding and Genetics Punjab Agricultural University, Ludhiana 141004, India; 11 Department of Botany, Doon College of Agriculture, Science and Technology, Camp Road, Selaqui, Dehradun 248011, India; 12 Department of Agronomy, Agricultural College and Research Institute, Tamil Nadu Agricultural University, Coimbatore 641003, India; 13 Department of Agronomy, SRM College of Agricultural Sciences, SRM Institute of Science and Technology, Chengalpattu 603201, India; 14 Department of Agronomy, S. Thangapazham Agricultural College, Vasudevanallur, Tenkasi 627760, India; 15 Department of Agricultural Entomology, SRM College of Agricultural Sciences, SRM Institute of Science and Technology, Chengalpattu 603201, India; 16 Physiology and Biochemistry Research Laboratory, Department of Biology, Science Faculty, Selcuk University, 42130 Konya, Turkey; 17 Clinic for Conservative Dentistry and Periodontology, School of Dental Medicine, Christian-Albrecht’s University, 24105 Kiel, Germany; 18 Centro Tecnológico de la Carne de Galicia, Rúa Galicia n◦ 4, Parque Tecnológico de Galicia, San Cibrao das Viñas, 32900 Ourense, Spain; 19 Área de Tecnología de los Alimentos, Facultad de Ciencias de Ourense, Universidad de Vigo, 32004 Ourense, Spain

**Keywords:** *Acacia catechu*, phytochemicals, bioactivities, natural antioxidant, antimicrobial

## Abstract

With the advent of pandemics and infectious diseases, numerous research activities on natural products have been carried out to combat them. Researchers are investigating natural products for the treatment and/or management of various infectious diseases and/or disorders. *Acacia catechu* (L.f.) Willd. belongs to the family Fabaceae (subfamily Mimosoideae) known as Khair or Cutch tree, possesses diverse pharmacological actions, and has been widely used in Asia and different parts of the world. The purpose of the present study is to highlight the phytochemical profile of different parts of *A. catechu*, the different biological activities of *A. catechu* extract, and the utilization of *A. catechu* as food and beverage. The present work constitutes a review of *A. catechu*; we performed searches (books, Google, Google Scholar, and Scopus publications) to compile the work/investigations made on *A. catechu* to the present. From our survey, it was concluded that the main phytochemicals compounds in *A. catechu* are protocatechuic acid, taxifolin, epicatechin, epigallocatechin, catechin, epicatechin gallate, procyanidin, phloroglucin, aldobiuronic acid, gallic acid, D-galactose, afzelchin gum, L-arabinose, D-rhamnose, and quercetin. The whole plant of *A. catechu* possesses a comprehensive variety of medicinal potentials such as antimicrobial, antidiarrheal, antinociceptive, antihyperlipidemic, antiulcer, antioxidant, antidiabetic, antiproliferative, haemolytic, and anti-inflammatory properties due to the presence of bioactive compounds like flavonoids, alkaloids, and tannins. However, even though the plant’s metabolites were reported to have many different pharmacological uses, there is limited information about their toxicity or clinical trials. Further research on diverse metabolites of *A. catechu* should be carried out to ensure the safety or utilization of this plant in the pharma or food industries and in the development of potent plant-based drugs.

## 1. Introduction

**Figure 1:**
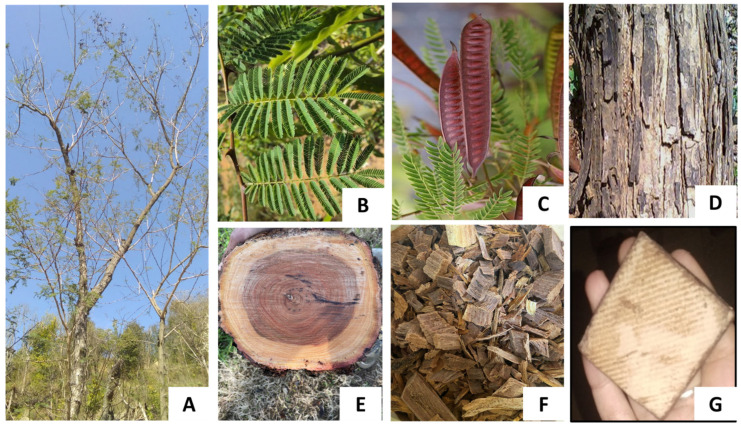
Different parts of *Acacia catechu*; (**A**) whole tree, (**B**) leaves, (**C**) fruits, (**D**) bark, (**E**) transverse section of wood, (**F**) heartwood chips, (**G**) concentrated extract shown in this figure.

**Figure 2:**
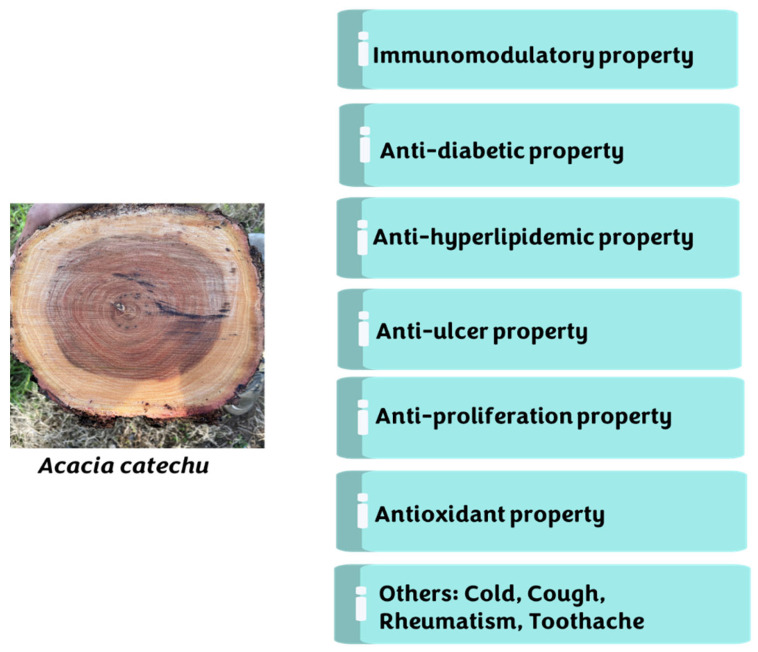
Overview of benefits/properties of *Acacia catechu*.

*Acacia catechu* (L.f.) Willd. is a deciduous tree belonging to the family Fabaceae and has been widely used in Ayurveda for many years for the treatment and prevention of various diseases and/or disorders. It is called Cutch tree in English, Kahir in Hindi and Khadira in Sanskrit [1,2] (Figure 1). Most people use boiled water of *A. catechu* (khair) for drinking purposes. The heartwood of this plant is a medicinally potent product known as Katha, having a wide range of therapeutic potential applications. Katha is made up of concentrated extracts of 10–20 years old *A. catechu* tree and is used as an ingredient in paan (betal leaf masticatory) and gives a red colour to saliva [3]. On chemical analysis of *A. catechu*, it was concluded that different parts contain very high amounts of flavonoids, tannins, and phenolic compounds, especially catechin/epicatechin, epigallocatechin, taxifolin, procyanidin, quercetin, taxifolin, etc. These active compounds such as catechin or epicatechin perform significant functions as an anti-inflammatory and antioxidant agent; likewise, tannins are found to be responsible for astringent action ions in the human body and have been considered as having good potential for curing wounds in the human body [4]. More specifically, catechin is abundant in this plant and serves as an antioxidant and antimicrobial agent [5,6]. Catechin is a 3,3′,4′,5,7-pentahydroxyflavan with two steric forms of (+)-catechin and its enantiomer. Its name is derived from the word “catechu”, used to describe the extract of *A. catechu*. In addition, catechin is also the name of the chemical family for all the compounds that are derived from catechin [7]. Numerous researchers have studied the pharmacological properties of extracts made from different parts of *A. catechu* (involving heartwood, bark, leaves, seeds, and pods) in recent years [8,9,10,11,12,13] (Figure 2). Different parts of *A. catechu* are depicted in Figure 1. The extract of *A. catechu* also exhibits numerous pharmacological activities, including immunomodulators [9,10], anti-hyperglycemic [11,12], antinociceptive [11], antihyperlipidemic [12], and antiulcer [13]. The flavonoids found in the hardwood of *A. catechu* have been utilized in a range of pharmaceutical activities, particularly in Asia, such as antibacterial, anti-cancer, anti-inflammatory, anti-viral, and cardiovascular reasons [14,15]. In a study, aqueous and ethanolic heartwood extracts of *A. catechu* were investigated for their immunomodulatory activity in a Swiss albino male [10]. Similarly, in another study, a root extract (95% ethanolic and aqueous extract) of *A. catechu* was studied for its antiulcer effect [13]. Likewise, it was determined that ethanolic bark extracts of *A. catechu* possess anti-diabetic properties, ascribed due to the inhibition of α-glucosidase and α-amylase activity [16]. In a research investigation, it was determined that both aqueous and methanolic extracts possess significant oxidative stress-modulating activity (using, 2,2-Diphenyl-1-Picrylhydrazyl (DPPH), ferric reducing antioxidant power (FRAP), 2,2′-Azinobis-(3-Ethylbenzothiazoline-6-Sulfonic Acid) (ABTS) and Superoxide Radical Scavenging Assay), and have the potential to be used as an antioxidant agent [17]. The bark of *A. catechu* was also proved to be effective against the growth of cancer cell lines (antiproliferative activity) such as A549 lung, PC-3 prostate, MCF-7 breast, Hep-G2 liver, HeLa cervix, and IMR32 brain [2]. In an antimicrobial study, an *A. catechu* extract (ethanolic and aqueous) was found to be effective against *Escherichia coli*, *Staphylococcus aureus*, *Salmonella typhi*, *Klebsiella pneumonia*, *Shigella sonnei* [16,18], and *Streptococcus mutans* [19]. An *A. catechu* ethanolic extract was tested in vitro for antifungal activity against three human pathogenic fungi: *Epidermophyton floccosum*, *Trichophyton rubrum*, and *Microsporum gypseum.* Other studies and some brief information about the tests are described in Section 4. The present paper is a comprehensive review of the literature (2000–2022) on the significant insights of chemical constituents, the presence of catechin and other flavonoids, indigenous uses, and the biological activities of *A. catechu*, in detail.

## 2. Phytochemical Constituents of *Acacia catechu*

In the heartwood of *A. catechu*, catechin content was 3.30% [20], 66.9% [21], and 3.580% [22,23]. Camphor (methanol extract), phytol (methanol extract), vitamin E acetate, 2-ethyl-3-methyl-1-butene, butylphosphonic acid, di(4-methoxybenzene (hydroethanolic extract), ellagic acid, quercetin, rutin, and kaempferol are present in the leaves [24], and the bark shows the presence of catechin (methanol extract), quercetin, kaempferol (ethyl acetate), kaempferol (methanol extract) ascorbic acid, riboflavin, thiamine, niacin, and carotenoids [24]. Gayathari et al. [20] and Bhardwaj et al. [22] showed the presence of catechin, rutin, isorhamnetin, and epicatechin in heartwood. Saha et al. [25] identified 41 compounds in ethanolic leaf extracts through the GC/MS technique. Li et al. [26] identified two new phenolic compounds viz. 5-hydroxy-2-[2-(4-hydroxyphenyl) acetyl]-3-methoxylbenzoic acid and (2S,3S)-3,7,8,30,40-pentahydroxyflavane in aqueous catechu extract through HPLC. The proximate and phytochemical composition of individual parts of *A. catechu* is described in Table 1.

**Figure 3:**
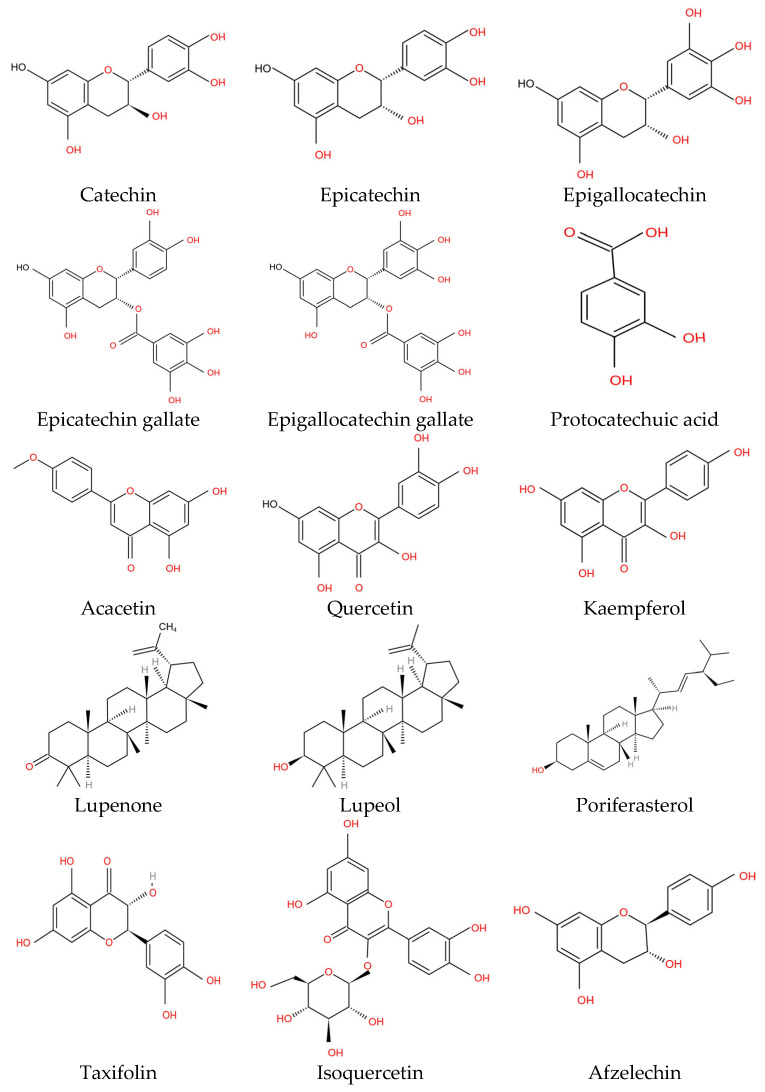
Structure of some major bioactive compounds of *Acacia catechu* [5,7,43].

Various pharmaceutical studies have shown that phytochemicals are beneficial to the prevention of illnesses [22]. The flavonoid class and subclass flavan-3-ols/flavonol are both referred to as catechin [33]. It is usually prominent in plants that have good antioxidant properties [10]. It has been found that *A. catechu* (heartwood portion) extract contains 23.1% epicatechin and 66.9% catechin [21]. *Acacia catechu* contains catechu tannic acid, catechuic acid (25% to 33%), catechu red, acacatechin, quercetin (10% to 12%), catechin (2% to 12%), epicatechin (25–33%), quercitrin, fisetin, gummy matter, phlebotannin, quercetin, tannins, cyanodol, polyphenols, and moisture [34,35]. Steroids, alkaloids, saponins, flavones, carbohydrates, glycosides, tannins, and phenolic compounds were found in the leaves of *A. catechu* [36]. Flavonoids are the most abundant class of naturally occurring phenolic chemicals, and they are found in a large variety of plant components, both free and as glycosides [37]. Anti-arthritic, antiangiogenic, antibacterial, anticancer, antiulcer, mitochondrial adhesion inhibition, protein kinase inhibition, and other biological actions have been demonstrated. In animal models, plant flavonoids inhibit tumour development [31,38]. Flavonoids also help decrease the threat of coronary artery disease by curing the oxidation of low-density lipoproteins (LDLs), lowering blood platelet coagulability, and increasing coronary vasodilation [39]. A study concluded that epigallocatechin gallate (EGCG), a major catechin, strongly inhibits the telomerase enzyme, which is required for cancer cells to proliferate by maintaining the tips of their chromosomes. As a result, it is possible that this is another reason for catechins’ anticancer properties [40,41]. Epicatechin is a white powder that has no odour. Epicatechin is a flavonol that belongs to the flavonoids category. Epicatechin is a powerful antioxidant that acts as an insulin mimic and, as a result, acts as an antidiabetic agent and also enhances heart function. It is used to treat diabetes mellitus. Epicatechin 3 gallate is used to treat dental caries and periodontal infections [42]. Various health benefits of the bioactives of *A. catechu* are discussed in Section 4. Some phytochemical compounds are shown in Figure 3.Major compounds isolated from A. catechu are mentioned in Table 2.

## 3. Medicinal Uses of *Acacia catechu*: Traditional Knowledge and Ayurveda

*Acacia catechu* is extremely valuable due to its therapeutic properties. This plant has long been used in traditional medicine, especially in Asia for its valuable nutritional properties [44]. This plant has the potential to produce functional foods and medications [24]. The *Acacia catechu* plant has been used traditionally to treat asthma, bronchitis, cancer, chest pain, diarrhea, mouth sores, sore, throats, ulceration, vitiligo, and wound healing and possesses antifungal, antiviral, spasmolytic, and hypoglycemic properties [13]. In Ayurveda, there are many references to prove that *A. catechu* is a beneficial plant with numerous curative properties [45] including for skin disease. Khadirarishta is a popular ayurvedic skin tonic made from the bark and heartwood of *A. catechu*. Additionally, heartwood extract has been utilized in many pharmaceutical products, including Lavangadi Vati [10]. “Ercha” a traditional Chinese drug used to treat coughs, diarrhea, skin ulcers, and blemishes [46] is prepared from this plant. The benefits of khoyer, according to folk medicine practitioners in Bangladesh, is used for pain relief and blood sugar control in diabetics [11]. This plant is widely used as chew sticks in different parts of the world because of its potential antibacterial properties [4,47]. The medicinal uses of *A. catechu* are described in Table 3.

## 4. Biological Activities of *Acacia catechu*

Numerous studies have been conducted in recent years to examine the pharmacological activities of *A. catechu* extract. In this review, we have discussed the in vivo and in vitro studies that included the immunomodulatory, antihyperglycemic, hepatoprotective, antidiarrheal, antinociceptive, antihyperlipidemic, antiulcer, antioxidant, antidiabetic, antiproliferative, antibacterial, antifungal, haemolytic, and anti-inflammatory properties of *A. catechu*.

### 4.1. In-Vivo Activities (Table 4)

#### 4.1.1. Immunomodulatory Activities

Many infectious diseases’ pathophysiology is influenced by the immune system. Immune responses can be modulated to treat such diseases. Immunomodulation is used to manage diseases, and it acts as natural substances which are derived from plants [62]. Sunil et al. [10] studied the immunomodulatory activity of aqueous and ethanolic heartwood extract at dosages of 100 mg/kg and 200 mg/kg in Swiss albino male mice and performed three different tests involving delayed-type hypersensitivity, hemagglutinating antibody titer, and plaque-forming cell (PFC) assay. The antibody titer is enhanced by heartwood extracts and stimulatory effect on the humoral response to SRBC. In the spleen, antibody-producing cells were determined by a plaque-forming cell assay. An activated humoral immune response was also indicated by a significant increase in antibody-producing cells in the spleen. There is a significant reduction in DTH responses after extract treatment, which indicates that the immune response to SRBC is chiefly due to the occurrence of high antibody titers rather than a generalized stimulation of immune responses involving T-cells. Plaque-forming cells revealed a significant result and had the highest number in ethanol extract at a dose of 200 mg/kg, i.e., 535.67 PFC/10^6^ spleen cells.

A similar study has been conducted to investigate the immunostimulatory activity of *A. catechu* by using swiss albino mice animal model. The fractioned heartwood extract in butanol, chloroform, and ethyl acetate at concentrations of 200 and 400 mg/kg b.w. was used to check the immunomodulatory activity. The authors used three tests: delayed-type hypersensitivity, hemagglutinating antibody titer, and plaque-forming cell (PFC) assay. The hemagglutinating antibody titer assay was used to determine the outcome of different fractions of extract. At a dose of 400 mg/kg b.w, a significant increase in the antibody titer and plaque-forming cells in mice blood was observed in butanol fraction at a rate of 512 PFC/10^6^ spleen cells and 499.67 PFC/10^6^ spleen cells, respectively. The DTH response was considerably increased in the treated group after 24 h of induction with the butanol fraction at a dosage of 400 mg/kg, which induce a great increase in paw thickness (3.41 mm). Similarly, aqueous extract doses at low ALD (5 mg/kg) and high AHD (50 mg/kg), were administered orally. For this, albino wistar rats were used. The effect was shown in a mouse lethality test, serum immunoglobulin levels, carbon clearance assay, neutrophil adhesion test, cyclophosphamide-induced neutropenia, and hemagglutination test. The effect of the extract on cell-mediated immunity was demonstrated in nylon fibers by a rise in neutrophil adherence. The phagocytic index was largely increased and a considerable shield adjacent to cyclophosphamide-induced neutropenia. *A. catechu* extract caused a considerable increase in serum immunoglobulin levels, decreased the mortality ratio in mice, and increased hemagglutination titer values. This shows that it has an effect on the humoral arm of the immune system. As a result, the aqueous extract has a considerable outcome on cell-mediated and humoral immunity [9]. However, from the results of the above studies, it was concluded that *A. catechu* could be used as an immunomodulating agent.

#### 4.1.2. Antihyperglycemic Activities

Diabetes mellitus is a grouping of metabolic conditions indicated by elevated blood glucose levels caused by insulin secretion abnormalities. Insulin is a hormone generated by the pancreas and regulates blood glucose levels in most people. Plant-based drugs are used to cure diabetic diseases and are of great interest as they have proven their hypoglycemic efficacy to varying degrees. The effect was explored in *A. catechu* by administering hydroethanolic leaf extracts orally for 30 days at doses of 200 mg/kg and 400 mg/kg concentrations in diabetic rats. The results indicate that the extract lowered blood glucose levels significantly. The activity of Aspartate aminotransferase, Alanine aminotransferase, and alkaline phosphatase in group III and group IV was found to be considerably decreased in rats as compared with the control group (Table 4) [12]. In diabetic rats, *A. ataxacantha* root extracts considerably reduced the activities of AST, ALT, and ALP [63].

In another study, the methanolic extract of four different doses (oral administration) was investigated for its antidiabetic effect on mice. The results revealed that methanolic extract had a dose-dependent and antihyperglycemic impact when used as a treatment on glucose-loaded mice. The drop in serum glucose levels was 28.6%, 35.9%, 37.0%, and 37.7% at extract doses 50, 100, 200, and 400 mg, respectively. At 400 mg extract, the effect was significant [11]. In a new investigation, an ethanolic extract and water-insoluble fraction of *A. catechu* were studied. The hypoglycemic activity of the ethanol extract and its component was considerable and dose-dependent [64]. The hypoglycaemic effect of the ethyl acetate extract was studied in non-diabetic and alloxan-induced diabetic albino rats. In the control group, the test drug at a dose of 500 mg/kg significantly lowered blood glucose levels after 2 h. In alloxan-induced diabetic rats, a dose of 250 and 500 mg/kg/day of *A. catechu* and the conventional treatment with 0.5 mg/kg/day of glibenclamide was extremely significant compared to the control group after seven days [60]. From the observations, it was concluded that *A. catechu* can be used for the production of supplementary or functional foods, reducing the risk of diabetes.

#### 4.1.3. Antihyperlipidemic Activity

Cholesterol is a necessary component of the cell membrane, as it ensures the adequate permeability and fluidity of the membrane. A higher total cholesterol level increases the risk of heart disease and stroke. Triglycerides are neutral fats that serve as an energy source and dietary fat transporter in the body. HDL is a high-density lipoprotein that reduces the risk of heart disease and stroke. Because it transports cholesterol from peripheral tissues to the liver, it is considered beneficial. While it deposits in blood vessel walls, (LDL) low-density lipoprotein is considered harmful cholesterol. LDL cholesterol is a strong analyst of coronary artery disease and atherosclerosis. The liver produces lipoprotein that is VLDL, and it serves as the internal lipid transport mechanism in the body. An *A. catechu* hydroethanolic leaf extract was given to the diabetic rat for 30 days at concentrations of 200 mg/kg and 400 mg/kg. Triglyceride, LDL, serum total cholesterol, and VLDL cholesterol were observed to be considerably higher in diabetic rats. Upon comparison of the diabetic to the normal rats, the amount of HDL serum was considerably lower. The flavonoids in the plant extract are responsible for boosting HDL levels [12]. The root extract of *A. ataxacantha* improved serum HDL levels and effectively reduced triglyceride serum, total cholesterol, VLDL, and LDL [63].

#### 4.1.4. Antiulcer Activity

The most frequent type of ulcer in humans is peptic ulcer. It is also known as Ulcus pepticum, a peptic ulcer disease in the gastrointestinal region that is typically acidic and really painful. *Helicobacter pylori* are spiral-shaped bacteria that dwell in the acidic and are responsible for around 80% of ulcers. *A. catechu* root extract was investigated in wistar albino rats utilized in 95% ethanolic and aqueous extract by measuring values in three tests, i.e., acute toxicity studies, NSAIDs-induced ulcer model, and Aspirin + Pylorus ligation-induced. For this, the animals fasted for 24 h before the experiment and were given free access to drinking water. The ulcer model generated by NSAIDs had the inhibitor effect. Animals were placed in ten groups containing six animals: group I and II had given 150 mg/kg ranitidine and 1 mL vehicles; groups III and IV were given a complete ethanolic root extract at 200 and 400 mg/kg doses; groups V and VI were given aqueous root extract at 200-400 mg/kg doses orally. The *A. catechu* ethanolic extract significantly shows the inhibition of 31.33% and 55.35% at 200 and 400 mg/kg, respectively. Additionally, an aqueous extract shows an inhibition equal to 54.54% and 71.14% at 200 and 400 mg/kg, respectively. The aqueous extract of *A. catechu* provided considerable protection against ethanol-induced ulcers and decreased the ulcer index [13]. *Acacia senegal* (Arabic gum) in adult male rat animal model showed effective protection against peptic ulcers. The results show that Arabic gum significantly reduces peptic ulcers [65].

#### 4.1.5. Antinociceptive Activity

In this activity, an *A. catechu* methanolic wood extract (at doses: 50, 100, 200, and 400 mg per kg b.w.) in Swiss albino mice was investigated for its antinociceptive effect. By intraperitoneal acetic acid administration in a dose-dependent and substantial method, the extract reduced writhing in mice. The percentages of writhing reduction were 51.5, 57.6, 63.6, and 69.6% in all four doses. The results show that 200 and 400 mg/kg b.w. of extract relieved pain better than aspirin. The current study reviled that the extract used for pain alleviation was also validated [65]. An *Acacia nilotica* aqueous extract, at a dose of 50 mg/kg of bark, caused a significant reduction in pain [66].

### 4.2. In-Vitro Activities (Table 5)

#### 4.2.1. Antidiabetic Activities

Hyperglycemia is a significant cause of diabetes mellitus (DM) and inhibitors namely carbohydrate-hydrolase such as α-glucosidase and α-amylase which provide an efficient technique for lowering postprandial hyperglycemia by controlling dietary carbs and glycogen breakdown. To know the effective values of *A. catechu*, Aryal et al. [16] investigated the antidiabetic potential of ethanolic bark extract, via α-glucosidase and α-amylase inhibition assays. Both assays demonstrated a good anti-diabetic impact; however, the α-amylase inhibition assay demonstrated the greatest inhibitory effect. As a consequence, the crude ethanol extract demonstrated anti-diabetic efficacy in α-amylase assays, with an IC_50_ of 67.8 µg/mL compared to acarbose (IC_50_ = 6.1 µg/mL). This is briefly explained in Table 5. In a similar study, both α-glucosidase and α-amylase inhibition assay against the ethanolic seed extract of *A. catechu* was evaluated. Both assays demonstrated a good anti-diabetic effect, although the α-glucosidase assay had the greatest inhibitory impact. At a dosage of 500 µg/mL, the inhibitory effect of *A. catechu* ethanolic extract of α-glucosidase was 53.77%. Furthermore, the IC_50_ value for inhibiting α-glucosidase activity was 187.80 µg/mL. α-glucosidase activity is higher than that of α-amylase. Because of the number of flavonoids and polyphenols in *A. catechu*, it has a high potential for blocking carbohydrate hydrolases [67].

#### 4.2.2. Antiproliferative Activities

In a study, bark extracts/fractions of *A. catechu* were investigated for their antiproliferative effect against six human cancer cell lines, i.e., A549 lung, PC-3 prostate, MCF-7 breast, Hep-G2 liver, HeLa cervix, and IMR32 cell line [2]. From the results, it was determined that the ethyl alcohol bark fraction (EAFB) was found to be effective against the growth of the MCF-7 cell line; the IC_50_ values of various cell lines were 137.5 μg/mL (MCF-7), 153.23 μg/mL (A549), 186.19 μg/mL (PC-3), 204.67 μg/mL (HepG2), 163.97 μg/mL (HeLa), and 251.33 μg/mL (IMR32). In methanolic fraction (MEB), the A549 cell line was determined to be the most effective with an IC_50_ value of 184.52 μg/mL. In *n*-butanol (NBFB) and aqueous bark fraction (AFB), the HeLa cell line was determined to be the most effective, having an IC_50_ value equal to 186.51 μg/mL and 241.30 μg/mL, respectively. In another study, an *A. catechu* heartwood methanolic extract (70%) (ACME) was tested against the breast carcinoma MCF-7 cell line. The cell viability percentage of the *A. catechu* heartwood extract at different concentrations (1 ng, 10 ng, 100 ng, 1 μg, 10 µg, and 100 µg) was (13.63879%, 16.88865%, 22.69579%, 32.92488%, 39.21151%, and 47.46937%) was observed. Furthermore, the MTT assay clearly demonstrates that ACME has a potent antiproliferative effect on breast cancer cells line with an IC_50_ value equal to 105.35 μg/mL [68]. Diab et al. [69] assessed the cytotoxicity of *A. catechu* plant extracts at various concentrations ranging from 10–100 μg/mL for 48 h. Compounds were investigated using sulforhodamine blue (SRB) and MTT assays. Nine different human cancer cell lines, i.e., lung cancer (A549), prostate cancer (PC-3), colon cancer (HCT-16 and Colo-205), breast cancer (T47D and MCF-7), and leukaemia (THP-1, HL-60, and K562) was used. The most effective cell line was found to be A549, PC3, MCF-7, HCT-16, and HL-60 with an IC_50_ value of 9.7 μg/mL, whereas the less effective cell line was observed to be T47D, Colo-205, THP-1, and K562 cells with an IC_50_ value of 37.8 μg/mL. Similarly, in another study, an *A. catechu* seed extract showed inhibitory effects against human SCC-25 oral carcinoma cells [70]. The MTT test was used to assess the antiproliferative impact of the *A. catechu* seed extract in SCC-25 cells. The proliferation of oral squamous carcinoma cells is significantly inhibited after a 24-h treatment with ACS extract. The highest dose of 1000 μg/mL of ACS extract was used in this study. The maximal antiproliferative efficacy was discovered to be more than 80%. Moreover, *A. catechu* bark extract showed inhibitory effects on SCC-25 oral squamous carcinoma cells [70]. The MTT assay revealed that 24-h treatment with *A. catechu* bark extract inhibited the growth of SCC-25 cells. The maximal antiproliferative efficacy was discovered to be 83%. By using linear regression analysis, the IC_50_ of ACB extract on oral squamous carcinoma cells (OSCC) was estimated and determined to be 52.09 μg/mL. Moreover, 95% ethanolic extract of A. catechu was found to be effective against HL-60 cell line with IC_50_ value of 9.7 μg/mL [71]. 

#### 4.2.3. Antioxidant Activities

*A. catechu* plant extracts have significant antioxidant potential, evaluated by a DPPH test [2]. It was observed that ethyl acetate bark fraction (EAFB) exhibited the highest scavenging ability, whereas the aqueous fraction of bark (AFB) had the lowest. In the ABTS assay, EAFB had the highest ABTS radical, followed by bark methanol extract (MEB), *n*-butanol bark fraction (NBFB), and AFB. In terms of ferric ion reduction potential, EAFB was the most effective, followed by MEB, NBFB, and AFB. In the CUPRAC test, EAFB had the highest reducing activity while AFB had the lowest. In the superoxide radical scavenging (SRS) experiment, EAFB exhibited the highest scavenging potential, whereas AFB had the lowest, and MEB and NBFB exhibited no significant differences. In the peroxyl radical scavenging (PRS) experiment, EAFB showed the lowest IC_50_ value, followed by MEB, NBFB, and AFB. The difference between MEB and NBFB was indistinguishable. (This is given briefly in Table 5). In a study, methanolic extract showed a considerable scavenging activity as compared to the aqueous extract. In another study, the DPPH assay was used to study the scavenging activity. The concentration of DPPH was 0.2 mM in the reaction mixture and the test sample concentration was (5–100 µg/mL). Discolorations were measured spectrophotometrically at 517 nm. The IC_50_ value was found to be 7.11 μg/mL in the DPPH assay [68]. In a similar study, Aryal et al. [16] used *A. catechu* ethanolic extracts to measure the DPPH antioxidant potential. The IC_50_ value was compared to quercetin (6.3 μg/mL). The plant extract radical scavenging capacity was 23.76 μg/mL. In another study, Kumar et al. [30] used a DPPH assay to measure the free radical scavenging activity at various dosages (100 μg/mL, 10 μg/mL, 1 μg/mL, and 0.1 μg/mL) to demonstrate in-vitro action in methanolic extracts of *A. catechu*. From the results, it was calculated that the hexane fraction of *A. catechu* (AHF), the effective concentration (EC_50_), and % inhibition values were shown to be more effective (0.89% EC_50_) [72]. The IC_50_ (µg/mL) value was calculated using a regression equation. Using the DPPH and ABTS assays, the ability to donate hydrogen was tested. IC_50_ values for AHF were 49 μg/mL and 13 μg/mL in DPPH and ABTS assays, respectively. IC_50_ values were found to be 35 μg/mL and 25 μg/mL in FIRA and CUPRAC assays, respectively. SSA and PRSA had IC_50_ values of 41 μg/mL and 27 μg/mL, respectively. Patil and Modak [17] performed tests for DPPH, FRAP, ABTS, and a superoxide radical scavenging assay to evaluate the antioxidant potential of *A. catechu* extracts in different solvent systems. In DPPH, the aqueous extracts showed 36.75%, 45.38%, and 50.48%, and methanolic extracts showed 37.14%, 52.43%, and 61.19% scavenging potential. In ABTS, the aqueous extracts showed 17.60%, 39.38%, and 62%, and the methanolic extracts showed 21.86%, 44.69%, and 66.69%. In FRAP, the aqueous extracts showed 0.246%, 0.449%, and 0.653%, and the methanolic extracts showed 0.221%, 0.404%, and 0.638% antioxidant potential. In the superoxide radical scavenging assay, the aqueous extracts showed 13.79%, 23.23%, and 45.19%, and methanolic extract showed 26.32%, 40.01%, and 48.49% potential.

**Table 5:** IC_50_ values of antiproliferation, antidiabetic, and antioxidant activities of different parts of *Acacia catechu* using different in vitro assays.

Bioactivity	Part Used	Type of Solvent Extract	Allied or Related Assay Conducted	Cell Line	Key Results	Outcome	Ref.
**Antiproliferation** (**Amritsar, India**)	Bark	Ethyl acetate, methanol, *n*-butanol fraction, an aqueous fraction of bark	MTT assay	A549, PC3, MCF-7, Hep-G2, HeLa, IMR32	IC_50_—Ethyl acetate: 251.33 μg/mL in IMR32; Methanol: 184.52 μg/mL in A549; *n*-butanol: 186.51 μg/mL; Aqueous: 241.30 μg/mL	*A. catechu* bark ethyl acetate fraction was found to be a very effective agent	[2]
**Antiproliferation**	Heartwood	70% methanolic extract (1–100 µg)	MTT assay	MCF-7 cell line	IC_50_: 105.35 µg/mL	MTT assay clearly shows that *A. catechu* methanolic extract has potent anti-proliferative activities against breast cancer cells	[70]
**Antiproliferation** (**Hosur, Tamil Nadu, India**)	Seed	Ethanolic extract (1000 μg/mL)	MTT assay	SCC-25 cell line	--	*A. catechu* ethanolic seed extract found cytotoxic at lower concentrations induces apoptosis of SCC-25 cells	[7]
**Antiproliferative** (**Tamil Nadu, India**)	Bark	Ethanolic extract (1000 μg/mL)	MTT assay	SCC-25 cell line	IC_50_: 52.09 μg/mL	*A. catechu* ethanolic bark extract induced apoptosis of SCC-25 cells	[7]
**Antioxidant** (**Amritsar, India**)	Bark	Methanol, Ethyl acetate fraction, *n*-butanol fraction, Aqueous fraction of bark	(A) DPPH (B) ABTS (C) FRAP (D)CUPRAC (E) SRS (F) PRS	--	IC_50_—Methanol: 155.99–246.84 μg/mL; Ethyl acetate: 92.48–177.2 μg/mL; *n*-butanol: 194.27–263.80 μg/mL; Aqueous: 259.3–529.30 μg/mL	Ethyl acetate fraction of bark was effectively scavenging free radicals and then MEB, NBFB, and AFB	[2]
**Antioxidant activity** (**Gandaki Province, Nepal**)	Bark	Ethanolic extract (100 μL)	FRAP	--	IC_50_: 23.76 μg/mL	Ethanolic extracts of *A. catechu* showed good scavenging activity when compared with standard	[16]
**Antioxidant activity** (**Chitwan**)	Bark	Methanolic extract (30%)	FRAP	--	IC_50_: 7.11 µg/mL	*A. catechu* showed effective DPPH radical scavenging activity	[68,69]
**Antidiabetic** (**Gandaki Province, Nepal**)	Bark	Ethanol extract (20 μL)	(A) α-Glucosidase (0.5 μg/mL) (B) α-Amylase (1.5 μg/mL)	--	IC_50_—α-Glucosidase: 10.3 μg/mL; α-Amylase: 67.8 μg/mL	Ethanolic bark extract has a good inhibitory effect against α-amylase and α-glucosidase	[16]
**Antiproliferation** (**Jammu, India**)	Fruits	95% ethanolic extract (100 µg/mL)	--	HL-60 cell line	IC_50_: 9.7 μg/mL	*A. catechu* ethanolic extract arrest in K562 cells that is induced G2/M	[71]
**Antidiabetic** (**Hosur, Tamil Nadu**)	Seed	Ethanol (100, 200, 300, 400, 500 µg)	(A) α-Glucosidase (0.07 mg/mL) (B) α-amylase inhibitory activity (0.5 mg/mL)	--	IC_50_—α-Glucosidase: 187.80 µg/mL; α-amylase: 341.20 µg/mL	The ethanolic seed extract of *A. catechu* showed a good anti-diabetic effect because polyphenols were present in the plant extract	[67]
**Antioxidant**	Resins	Methanol extract (0.1–100 µg/mL)	DPPH	--	EC_50_: 0.89 µg/mL	*A. catechu* methanolic extract showed much effective antioxidant activity	[30]
**Antioxidant** (**Amritsar, India**)	Leaves	Hexane fraction	DPPH, SSA, FIRA, PRSA, CUPRAC, ABTS	--	IC_50_: 132.55–499.45 µg/mL	Hexane fraction showed potent antioxidant activity	[72]
**Antioxidant** (**Dapoli Krishi Vidyapeeth, Dapoli**)	Bark	Aqueous and methanolic extract	DPPH, FRAP, ABTS, SRS Lipid peroxidation	--	IC_50_—Aqueous: 48.65–52.18 mg/g; Methanolic: 49.65–54.44 mg/g	*A. catechu* showed significant activities in aqueous and methanolic bark extracts	[17]

IC_50_—Half-maximal inhibitory concentration; MTT—3-(4,5-dimethylthiazol-2-yl)-2,5-diphenyl-2H-tetrazolium bromide; DPPH—2,2-diphenyl-1-picryl-hydrazyl-hydrate; ABTS—2,2′-azino-bis (3-ethylbenzothiazoline-6-sulfonic acid; FRAP—Ferric reducing ability of plasma; CUPRAC—Cupric Reducing Antioxidant Capacity; SRS—Superoxide Radical Scavenging; PRS—Peroxyl Radical Scavenging; Cell line: A549—lung cancer; PC-3—Prostate cancer; HCT-16 and Colo-205—Colon cancer, T47D and MCF-7—Breast cancer, THP-1, HL-60, and K562—Leukaemia, SCC—Squamous carcinoma cells, Hep-G2—Liver, HeLa—Cervix, and IMR32—Brain.

#### 4.2.4. Antimicrobial Activities

*Acacia catechu* confirmed good to excellent antimicrobial activities, depending on the organisms (Table 6). In a study, *A. catechu* extracts were used against *Escherichia coli*, *Staphylococcus aureus*, *Salmonella typhi*, *Klebsiella pneumonia*, and *Shigella sonnei* through the agar well diffusion method [16,18]. The MIC and MBC of an *A. catechu* aqueous fraction of bark were 6.25 and 12.5 mg/mL, while the drug MIC and MBC were 0.0625 and 0.25 mg/mL. In another study by Joshi et al. [73], there was no activity against *S. paratyphi*, *E. coli*, *S. typhi,* and *Proteus mirabilis* in any of the extracts. Diethyl ether extract was used to assess the highest zone of inhibition (ZOI) against the *Pseudomonas* bacterial strain. Diethyl ether, methanol, and ethyl acetate extract demonstrated better inhibition whereas hexane and chloroform showed modest action. *Shigella* sp. and *Bacillus subtilis* showed good inhibitory effects in the ethyl acetate extract with a minimum bactericidal concentration (MBC) of 50 mg/mL and at 100 mg/mL (MBC). Another test performed by Geetha et al. [19] demonstrated the efficacy of ethanolic and aqueous extracts of *A. catechu* against microbial strains. From the results, it was observed that the ethanolic extract was more effective against *Streptococcus mutans*, with the ZOI being 24 mm diameter at conc. 200 µg and it was least effective against *Lactobacillus acidophilus*, with the ZOI being 16 mm at conc. 200 µg. Among the other bacterial species studied, *Streptococcus salivarius* showed a ZOI of 19 mm diameter (at conc. 200 µg). MIC and MBC in the ethanolic extract were found to be low as compared to the aqueous extract. The ethanolic extract had low MIC and MBC values in *Streptococcus mutans* and *Enterococcus faecalis* at 0.5 mg/mL and 1 mg/mL concentrations. At 2 mg/mL extract concentration, *Lactobacillus acidophilus* had a higher MIC and MBC value as compared to *Streptococcus salivarius* at 1 mg/mL. The lower the MIC and MBC value, the more efficient the extract is, whereas the higher the MIC and MBC, the less effective the extract is. A similar test was performed by Patel et al. [74] using different fractions (chloroform, methanol, and petroleum ether) of an *A. catechu* plant extract against *S. aureus*, *P. aeruginosa*, *E. coli,* and *Bacillus subtilis*. The maximum zone of inhibition was studied against *E*. *coli* (8.8 mm) in the methanol solvent. The highest MIC value was found in the petroleum ether against *P. aeruginosa* (MIC: 10 μg/mL), after that, in water in *B. subtilis* (MIC: 20 μg/mL), followed by chloroform in *S. aureus* (MIC: 30 μg/mL). Another investigation by Negi and Dave [27] demonstrated that methanolic extracts of *A. catechu* possess significant antimicrobial activity against different bacterial strains, i.e., *B. subtilis* (1000 μg/mL), *S. aureus* (1000 μg/mL), *S. typhi* (700 μg/mL), *E. coli* (1500 μg/mL), *P. aeruginosa* (2000 μg/mL), and *Candida albicans* (1500 μg/mL). Furthermore, methanol extracts were found to be the most inhibiting zone diameters ranging from 18 to 22 mm, followed by hexane and acetone extracts.

#### 4.2.5. Antifungal Activities

*Acacia catechu* ethanolic extract was tested in vitro for antifungal activity against three human pathogenic fungi: *Epidermophyton floccosum*, *Trichophyton rubrum*, and *Microsporum gypseum* [19]. For the study, the agar well diffusion method was used. The extract had no antidermatophytic activity, showing that it was ineffective. In another study, an *A. catechu* extract was assessed for its antifungal activity against *Fusarium oxysporium* [73]. The highest ZOI (17 mm) was calculated in the ethyl acetate fraction. Only *F. oxysporium* was shown to be active in the hexane fraction. Hexane had little activity against fungal organisms. Ethyl acetate and diethyl ether fraction showed high antifungal activity, and methanol and chloroform showed moderate activity. These results show that ethyl acetate extract has a strong antifungal activity while hexane extract has a negligible antifungal activity (Table 6).

Due to the exceptional health-promoting activities of *A. catechu,* they have applications in the food industry as ingredients and in the beverage industry. These applications are highlighted in Section 5.

## 5. Utilization of *A. catechu* as Food and Beverage as Supplement Due to Their Bioactive Compounds

*A. catechu* is a famous herb, used in traditional Chinese medicinal systems. Irrespective of the phytochemical profile and pharmacological benefits, the extracts or dried powder of *A. catechu* prepared from different parts of the plant have been used for many decades in different parts of the world. *Acacia catechu* is valued in India for making two commercially important products from its heartwood: Katha and cutch [75]. Katha is used as an ingredient of paan (betel leaf masticatory) which is used traditionally and as well as chewed after meals in South Asia. It is also used as a flavouring agent in ice cream, beverages, candy, and condiments. Cutch is used as a tanning agent for leather, dye, mail bags, etc., and in oil-well drilling as a viscosity modifier of drilling mud [76]. Scientists have been exploring natural plant products rich in alkaloids, flavonoids, steroids, phenolics, terpenes, volatile oils, etc., for use in medicines, cosmetics, dyes, foods, and as a flavoring agent. Flavonoids are one of the major secondary compounds which are important for humans due to their antioxidative and anticancer activities. Quercetin, present in the *A. catechu* plant, was found to have anti-inflammatory, antioxidant, and anticancer properties [77]. Catechin and epicatechin present in Katha are used as food supplements. They are powerful antioxidants that act as insulin mimics and, as an antidiabetic agent and also enhance heart function. They are used to treat diabetes mellitus. Epicatechin 3 gallate is used to treat dental caries and periodontal infections [42]. Although, there are not many research studies (in vivo and clinical trials) clarifying the safety and utilization of *A. catechu* as a food supplement. Thus, there is a need to further investigate the toxicity and safety aspects of *A. catechu* as a food product.

## 6. Materials and Methods

All information related to this plant was gathered from previously published data, including phytochemicals, indigenous uses, and in vivo and in vitro studies of different parts of *A. catechu*. All the reviewed articles were available as published work on online databases (Science Direct, PubMed, Web of Science, and Google Scholar) and were found through different key phrases (*Acacia catechu*, phytochemicals, bioactivities, natural antioxidant, and antimicrobial). Botanical names of plant species were validated from the online website www.theplantlist.org, (accessed on 29 September 2022).

## 7. Conclusions

The use of herbal medicines has become a global subject with medical and economic ramifications over the past few decades. *Acacia catechu* is a medicinal plant used for the treatment of various diseases in traditional and modern medicinal systems. The outcome of this study showed that *A. catechu* shows the presence of many bioactive compounds and displays polyphenolic and flavonoid contents, i.e., catechin, epicatechin, quercetin, lupeol, kaempferol, etc., in different parts (involving heartwood, leaves, bark, seed). The plant has been extensively studied for its pharmacological activities, and the results show that it has potent immunomodulatory, hypoglycaemic, hepatoprotective, antidiarrheal, antipyretic, antinociceptive, antihyperlipidemic, antiulcer, antioxidant, antimicrobial, antidiabetic, antiproliferative, antibacterial, antifungal, haemolytic, and anti-inflammatory properties that make this plant very valuable. However, this plant is helpful in curing various ailments. These data prove that *A. catechu* is a potential medicinal plant and could be used in the discovery or production of novel drugs. Although *A. catechu* has a high nutritional and phytochemical value, there is still a lack of research on its molecular mechanism and a need for chemical investigations and clinical trials to ensure the plant’s safety in the food and pharmaceutical industries.

## Figures and Tables

**Table 1:** Different Compounds are present in different parts of *Acacia catechu* in different extracts.

Plant Part	Type of Extract Used	Total No. of Compound Identified	Different Compounds Present in *Acacia catechu*	Technique Used	Ref.
**Leaves**	Methanol	2	Camphor, Phytol	GC/MS/flame-ionization	[23,27]
	Ethanol	10	Flavonoids—(+)-catechin, (–)-epicatechin, (+)-afzelechin, (−)-epiafzelechin, (+)-mesquitol, kaempferol, quercetin, quercetin 3-methyl ether, ellagic acid, and caryatin.	HPLC/Diode array	[28]
	Ethanol	2	Rutin, Quercetin	HPTLC/Densitometer or TLC scanner	[29]
	Methanol (Hexane fraction)	9	Polyphenolic—Gallic Acid, Catechin, Chlorogenic acid, Epicatechin, Coumaric acid, Rutin, Ellagic acid, Quercetin, Kaempferol	UHPLC/Photo diode array	[30]
	Methanol	4	Caprylic acid methyl ester, Lauric acid methyl ester, 2-Ethyl-3-methyl-1-butene and Myristic acid methyl ester	GC/MS/Mass detector	[23]
	Ethanolic	41	L-(+) lactic acid, L-alanine, L-valine, Urea, Pipecolic acid, Glycerol, Phosphoric acid, L-threonine, Glycine, Succinic acid, Glyceric acid, Beta-alanine, D-malic acid, O-acetylsalisylic acid, L-glutamic acid 3 (dehydrated), 4-guanidinobutyric acid, Phenylalanine, Phenylethylamine, Meleamic acid, L-glutamic acid, Lauric acid, L-asparagines, Xylitol, Arabitol, Putrescine, Methyl-beta-D-galactopyranoside, Quinic acid, Allantoin, Tyramine, D-sorbitol, D-mannitol, Gallic acid, Palmitic acid, Dopamine (hydroxytyramine), L-tryptophan, Stearic acid, Serotonin, Sucrose, (-)-epicatechin, Catechin, Isoquercitrin	GC/MS/Nuclear magnetic resonance	[25]
**Bark**	Methanol, Ethyl acetate	5	Polyphenolic—Catechin, Quercetin, Rutin, Kaempferol	UHPLC/Photo Diode Array	[2]
	Ethyl acetate and aqueous fraction	29	Catechin, Epicatechin, Gallocatechin, Epigallocatechin, Procyanidin B1, Procyanidin B3, Emodin, Afzelechin, Epiafzelechin, Maclurin, Irisflorentin, Naringenin, Isoquercetin, Diosmetin, Chrysin, Myricetin, Kaempferol, Avicularin, Prodelphinidin B3, Prodelphinidin B, Quercetin, Taxifolin, Acacetin, Aciculatinone, Gossypin, Pterocarpin, Isorhamnetin, Trihydroxy dimethoxyflavone	LC-HRMS/diode array	[16]
**Heartwood**	Ethanol, methanol, petroleum ether	5	Gallic acid, Protocatechuic-acid-4-glucoside, Quercetin, 3-rhamnoside, Quercetin 3-glucuronide, Epicatechin	HPLC/Photo Diode Array	[31]
	Methanol	1	Catechin	UV-Visible Spectrophotometer	[32]
**Catechu**	aqueous	2	Phenolic compound—rhamnetin, 4-hydroxyphenol, 3,3′,5,5′,7-pentahydroxyflavane, fisetinidol, 5-hydroxy-2-[2-(4-hydroxyphenyl) acetyl]-3-methoxylbenzoic acid, (2S,3S)-3,7,8,30,40-pentahydroxyflavane	HPLC/UV spectrophotometer	[26]
**Seed**	Methanol	2	Catechin, Quercetin	HPLC/UV detector	[1]

HPLC: High Performance Liquid Chromatography, HPTLC: High Performance Thin Layer Chromatography, UHPLC: Ultra High-Performance Liquid Chromatography, GC-MS: Gas Chromatography—Mass Spectrometry, LC/HRMS: Liquid Chromatography—High Resolution Mass Spectrometry, LC-MS/MS: Liquid Chromatography with Tandem Mass Spectrometry, UV-Visible Spectrophotometer: Ultra Violet-Visible Spectrophotometer.

**Table 2:** Major compounds, molecular formula, and classes of the compounds in *Acacia catechu*.

Compounds	Molecular Formula	Classes of Compounds
**Catechin**	C_15_H_14_O_6_	Flavonoids
**Epicatechin**	C_15_H_14_O_6_	Flavonoids
**Epigallocatechin**	C_15_H_14_O_7_	Flavan
**Epicatechin gallate**	C_22_H_18_O_10_	Flavonoids
**Epigallocatechin gallate**	C_22_H_18_O_11_	Flavonoids
**Protocatechuic acid**	C_25_H_48_O_4_Si_3_	Hydroxybenzoic acid derivatives
**Acacetin**	C_16_H_12_O_5_	4′-o-methylated flavonoids
**Quercetin**	C_15_H_10_O_7_	Flavonoids
**Kaempferol**	C_15_H_10_O_6_	Flavonols
**Lupenone**	C_30_H_48_O	Triterpenoids
**Lupeol**	C_30_H_50_O	Triterpenoids
**Poriferasterol**	C_29_H_48_O	--
**Taxifolin**	C_15_H_12_O_7_	Flavanonols
**Isoquercetin**	C_21_H_20_O_12_	Flavonoid-3-o-glycosides
**Afzelechin**	C_21_H_20_O_12_	Flavan-3-ol

**Table 3:** Traditional uses of *Acacia catechu*.

Part Used	Disorder/Use	Types of Remedy	Method of Administration	Ref.
**Root**	Ulcer	Paste	Make a paste of root and apply on ulcer	[48]
**Root**	Mouth ulcer	Paste	Make a paste of root and apply on mouth	[49]
**Root**	Tuberculosis	Paste	For tuberculosis, two spoonfuls of root paste were administered orally on an empty stomach every day for 60 days	[50]
**Root**	Rheumatism and toothache	Paste	Make a paste of root and apply on mouth	[51]
**Bark**	Cold and cough	Decoction	To treat cold and cough, a decoction of bark combined with milk is used	[52]
**Bark**	Cold and cough	Extract	For three days, take two spoons of bark extract twice a day	[53]
**Bark**	Diarrhea	Decoction	To treat severe diarrhea, the bark decoction is used alone or in conjunction with opium	[9,52]
**Stem**	Diarrhea	Decoction	The stem is cut into small pieces, decocted, and the resulting decoction is drunk	[54]
**Bark**	Toothache	As such	In the cavity of a painful tooth, place the piece of bark	[55]
**Bark**	Skin disorder	Paste	Make a paste of bark and use it	[55]
**Bark**	Haemoptysis, gonorrhea.	Juice	Fresh bark juice has been taken	[56]
**Bark**	Leucorrhoea, menstrual complaints	Extract	Bark extract is used	[44]
**Bark**	Leprosy	Decoction	Bark decoction is given	[27]
**Bark**	Fracture bone, dislocation of bones, sprains	Decoction	For fractures, dislocations of bones, sprains, and cold, use a bark decoction in combination with *Scindapsus officinalis*	[57]
**Bark**	Asthma	Decoction	500 g of bark is decocted in 500 mL of cow’s milk with one teaspoon of sugar. For two weeks, one glass of this decoction is taken in the morning on an empty stomach	[58]
**Leaves**	Dysentery, gonorrhea	Extract	Extract of leaves is used	[13,44]
**Flower**	Dysentery	Extract	Extract of flower is used	[13]
**Seed**	Leukaemia	Extract	Seeds with a saline extract have leucoagglutinating activity against leukemic cells	[13]
**Gum**	Body pain	As such	Gum with sugar taken for 10–15 days	[59]
**Wood**	intestinal pain	Decoction	For intestinal pain, a decoction of wood is taken orally	[55]
**Heartwood**	Cure fever during pregnancy	Decoction	The decoction is made by boiling heartwood with other ingredients. Pregnant women drink it as tea to keep their bodies warm. It is also used to treat the fever of pregnant women	[52]
**Heartwood**	Body pain	Boiled	Boiled water of heartwood chips is used to take baths by women after delivery. It is useful to heal the body pains	[52]
**Katha**	Piles	As such	To treat piles, Katha is applied to a lemon slice and swallowed on an empty stomach on a daily basis	[52]
**Katha**	Mouth ulcer	As such	Katha is applied for mouth ulcers	[31]
**Heartwood**	Fever, diarrhea, leucorrhoea, piles, and erysipelas.	Extract	Heartwood extract called Katha is used for the treatment	[60]
**Katha**	Stomach ache	Pellet	Pellet of Katha is taken	[61]

**Table 4:** In vivo activities of different parts of *Acacia catechu*.

Biological Activity/Region	Type of Solvent Used for Extraction	Mode of Administration/Dosage	Experimental Model and Duration	Key Findings	Outcome	Ref.
**Antihyperglycemic (Kerala, India)**	Hydroethanolic leaf extract (200 and 400 mg/kg)	STZ-induced diabetic/Orally	Male albino rats (*n* = 42)/30 days	At a dosage of 200 and 400 mg/kg the values of AST, ALT, and ALP was 229.1, 57.5, and 293.85 U/L, and 211.23, 54.21, and 282.31 U/L, respectively.	At 200 mg/kg and 400 mg/kg concentrations, a hydroethanolic leaf extract showed a significant decrease in serum glucose levels in diabetic rats.	[12]
**Antihyperlipidemic (Kerala, India)**	Hydroethanolic leaf extract (200 and 400 mg/kg)	Orally	Male albino rats (*n* = 42)/30 days	At concentrations of 200 mg/kg and 400 mg/kg, the values of total cholesterol, triglyceride, HDL, LDL, and VLDL cholesterol were 124.66 mg/dL, 157.03 mg/dL, 25.48 mg/dL, 67.78 mg/dL, and 31.4 mg/dL, and 114.16 mg/dL, 152 mg/dL, 27.21 mg/dL, 56.55 mg/dL, and 30.4 mg/dL, respectively.	At 200 mg/kg and 400 mg/kg concentrations, hydroethanolic leaf extracts showed that total cholesterol, triglyceride, LDL, and VLDL cholesterol levels were significantly decreased, and HDL cholesterol levels significantly increased in rats.	[12]
**Antiulcer** **A) Absolute alcohol-induced ulcer model** **B) NSAIDs-induced ulcer model (Raipur Rani, Panchkula)**	Ethanol and aqueous extracts (200 and 400 mg)	Orally	Wistar albino rats (A) *n* = 20, B) *n* = 60)/24 h	(A) In the absolute alcohol-induced ulcer model the inhibition percentage was 67.87% (B) In the NSAIDs-induced ulcer model, the inhibition percentage was 71.14%.	*A. catechu* aqueous extract at a dose of 400 mg showed maximum percentage inhibition in the NSAIDs-induced ulcer model.	[13]
**Immunomodulatory** **(A) Hemagglutinating antibody titer** **(B) Plaque-forming cell (PFC) assay** **(C) Delayed type hypersensitivity (DTH)** **(Kannur District, Kerala, India)**	Aqueous (100–200 mg/kg) and ethanol (100–200 mg/kg)	Orally	Swiss albino male mice	(A) Antibody titer 117.33 HA titer (B) 535.67 PFC/10^6^ spleen cells (C) In aqueous extract at 100 mg/kg conc. the value was 1.05 mm and at 200 mg/kg conc. the value was 0.9783 mm. In ethanol extract at 100 mg/kg conc. the value was 0.2612 mm and for 200 mg/kg conc. the value was found 0.1842 mm	(A) Ethanol heartwood extracts on humoral immune response 200 mg/kg caused a significant rise in antibody titer. (B) Maximum number of plaque-forming cells (PFC) was observed in ethanol extract at 200 mg/kg. (C) The results showed that both the extracts (water and ethanol) at 100 and 200 mg/kg doses, significantly inhibited foot paw edema.	[10]
**Immunostimulatory** **(A) Hemagglutinating antibody titer** **(B) Plaque forming cell (PFC) assay** **(C) Delayed type hypersensitivity (DTH)** **(Kannur District, Kerala, India)**	Butanol, chloroform, and ethyl acetate fractions (500, 1000, 2000, and 4000 mg/kg b. w)	Orally	Swiss albino male mice (*n* = 54)/30 days	(A) 512 HA titer in the blood of mice (B) 499.67 PFC/10^6^ spleen cells (C) 3.41 mm	(A) Effect of heartwood butanol fraction on the humoral immune response at 400 mg/kg caused a significant rise in antibody titer. (B) Maximum number of plaque-forming cells was observed in butanol fraction at 400 mg/kg (C) In butanol fraction, DTH response was significantly increased in paw thickness at a dosage of 400 mg/kg b.w. after 24 h of induction.	[10]
**Antihyperglycaemic** **(Bangladesh)**	Methanolic extract (50, 100, 200, and 400 mg/kg b. w.)	2 g/kg body weight of glucose/Orally	Swiss albino mice (male) (*n* = 36)/2 h	37.7% lowering of serum glucose level	The study showed that *A. catechu* wood extract showed significant results in the methanolic extract at a concentration of 400 mg/kg b.w.	[11]
**Antinociceptive (Bangladesh)**	Methanolic extract (50, 100, 200, and 400 mg/kg b. w.)	Orally	Swiss albino mice (*n* = 36)/2 h	69.6% acetic acid-induced gastric pain	The study showed that methanolic wood extract of *A. catechu* showed significant results at a concentration of 400 mg/kg b.w.	[11]

AST: Aspartate aminotransferase, ALT: Alanine aminotransferase, ALP: Alkaline phosphatase, HDL: High density lipoprotein, LDL: low density lipoprotein, VLDL: Very low-density lipoprotein, NSAID: Non-steroidal anti-inflammatory drugs.

**Table 6:** Antimicrobial and antifungal activities of different parts of the *Acacia catechu* plant parts using agar well diffusion and disc diffusion methods.

Activity	Plant Part	Extract	Tested Strains	Key Results	Ref.
**Antifungal (Nepal)**	Heartwood	Ethyl acetate extract	*Fusarium oxysporium*	ZOI 17 mm	[73]
**Antimicrobial** (**Gandaki, Nepal)**	Bark	Aqueous fraction (50 mg/mL)	*Staphylococcus aureus*	MIC: 6.25 mg/mL ZOI: 14 mm	[16]
**Antimicrobial**	Resin part (Katha)	Methanol (1 mg/mL) Chloroform Petroleum ether Water (1 mg/mL)	*Staphylococcus aureus*	MIC:30 μg/mL	[74]
			*Escherichia coli*	MIC:60 μg/mL	
			*Pseudomonas aeruginosa*,	MIC:10 μg/mL	
			*Bacillus subtilis*	MIC: 20 μg/mL	
**Antimicrobial** (**Gandinagar, Gujarat**)	Leaves	Methanol	*Bacillus subtilis*	MIC: 1000 µg/mL	[27]
			*Salmonella typhi*	MIC: 700 µg/mL	
			*Staphylococcus aureus*	MIC: 1000 µg/mL	
			*Escherichia coli*	MIC: 1500 µg/mL	
			*Candida albicans*	MIC: 1500 µg/mL	
			*Pseudomonas aeruginosa*	MIC: 2000 µg/mL	
**Antimicrobial** (**Nepal**)	Heartwood	Diethyl ether extract	*Pseudomonas* species	ZOI: 15 mm	[73]

MIC—Minimum inhibitory concentration, ZOI—Zone of inhibition.

## Data Availability

Not applicable.
